# Mucosal Targeting of a BoNT/A Subunit Vaccine Adjuvanted with a Mast Cell Activator Enhances Induction of BoNT/A Neutralizing Antibodies in Rabbits

**DOI:** 10.1371/journal.pone.0016532

**Published:** 2011-01-27

**Authors:** Herman F. Staats, Jeffrey R. Fielhauer, Afton L. Thompson, Alice A. Tripp, Ashley E. Sobel, Massimo Maddaloni, Soman N. Abraham, David W. Pascual

**Affiliations:** 1 Department of Pathology, Duke University Medical Center, Durham, North Carolina, United States of America; 2 Department of Immunology, Duke University Medical Center, Durham, North Carolina, United States of America; 3 Department of Molecular Genetics and Microbiology, Duke University Medical Center, Durham, North Carolina, United States of America; 4 Human Vaccine Institute, Duke University Medical Center, Durham, North Carolina, United States of America; 5 Department of Immunology and Infectious Diseases, Montana State University, Bozeman, Montana, United States of America; University of Cincinnati College of Medicine, United States of America

## Abstract

**Background:**

We previously reported that the immunogenicity of Hcβtre, a botulinum neurotoxin A (BoNT/A) immunogen, was enhanced by fusion to an epithelial cell binding domain, Ad2F, when nasally delivered to mice with cholera toxin (CT). This study was performed to determine if Ad2F would enhance the nasal immunogenicity of Hcβtre in rabbits, an animal model with a nasal cavity anatomy similar to humans. Since CT is not safe for human use, we also tested the adjuvant activity of compound 48/80 (C48/80), a mast cell activating compound previously determined to safely exhibit nasal adjuvant activity in mice.

**Methods:**

New Zealand White or Dutch Belted rabbits were nasally immunized with Hcβtre or Hcβtre-Ad2F alone or combined with CT or C48/80, and serum samples were tested for the presence of Hcβtre-specific binding (ELISA) or BoNT/A neutralizing antibodies.

**Results:**

Hcβtre-Ad2F nasally administered with CT induced serum anti-Hcβtre IgG ELISA and BoNT/A neutralizing antibody titers greater than those induced by Hcβtre + CT. C48/80 provided significant nasal adjuvant activity and induced BoNT/A-neutralizing antibodies similar to those induced by CT.

**Conclusions:**

Ad2F enhanced the nasal immunogenicity of Hcβtre, and the mast cell activator C48/80 was an effective adjuvant for nasal immunization in rabbits, an animal model with a nasal cavity anatomy similar to that in humans.

## Introduction

**Figure 1:**
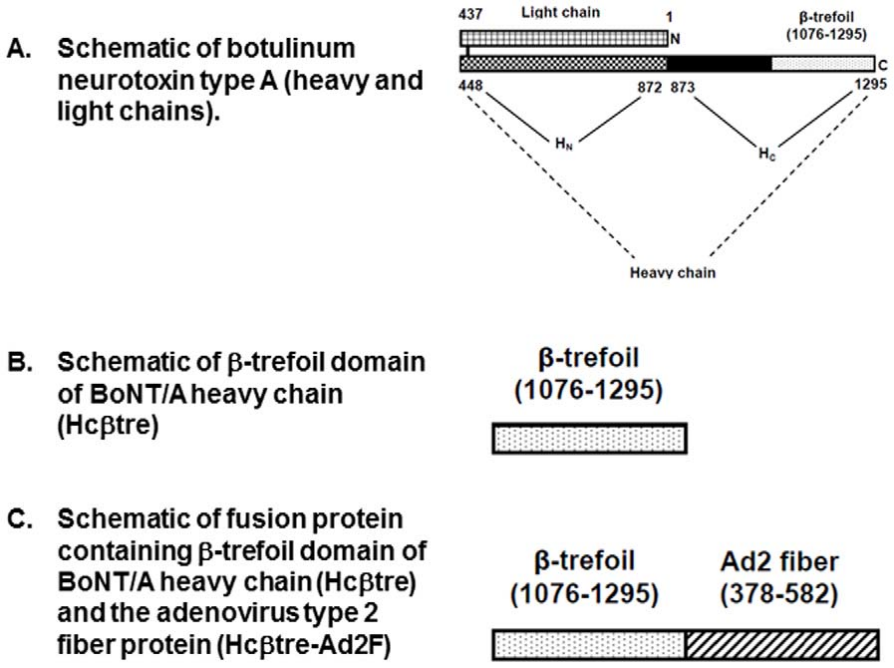
Schematic representation of Hcβtre-Ad2F fusion protein. **A**. schematic representation of botulinum neurotoxin type A (heavy and light chains). **B**. Schematic of β-trefoil domain of BoNT/A heavy chain (Hcβtre). **C**. Schematic of fusion protein containing β-trefoil domain of BoNT/A heavy chain (Hcβtre) and the adenovirus type 2 fiber protein (Hcβtre-Ad2F).

*Clostridium botulinum* is a spore-forming anaerobe which produces seven distinct neurotoxin serotypes (A–G). Botulinum neurotoxin is synthesized as a 150 kDa single chain protein and cleaved by proteases to yield a 100 kDa heavy chain (Hc) linked by a disulfide bridge to a 50 kDa light chain (Lc) [1], [2] (**Figure 1**). The Hc encompasses the neuronal cell binding β-trefoil domain [3] and membrane translocation units, and the Lc cleaves SNARE proteins, required for the release of acetylcholine at the neuromuscular junction. The botulinum neurotoxins (BoNT), combined with tetanus neurotoxin, comprise the class of clostridial neurotoxins. Clostridial neurotoxins are the most poisonous natural substances known to man; oral consumption of as little as 7 µg or inhalation of 700 ng is predicted to be lethal to a 150 lb individual [4].

A toxoid vaccine composed of formalin inactivated botulinum neurotoxin has been used as the botulinum neurotoxin vaccine for decades [5], [6]. The declining immunogenicity of the toxoid vaccine and the availability of molecular biology techniques to produce non-toxic subunit immunogens has lead to the development of next generation botulinum vaccines that are based on recombinant fragments of the heavy chain [5]. A recombinant botulinum vaccine based on the cell binding domain (Hc) is currently being tested in human clinical trials (http://clinicaltrials.gov and [5]).

We previously reported that a recombinant immunogen containing botulinum neurotoxin type A (BoNT/A) Hc β-trefoil domain (Hcβtre) induced complete protection against a 20,000 LD_50_ BoNT/A challenge in mice when used as a nasal vaccine immunogen coadministered with cholera toxin as a mucosal adjuvant [7]. Additionally, production of a fusion protein immunogen that contained the Hcβtre fused to the adenovirus type 2 fiber protein as a mucosal targeting ligand (Hcβtre-Ad2F) exhibited superior immunogenicity when compared to the Hcβtre subunit immunogen after nasal (or parenteral) immunization of mice while also inducing complete protection against a 20,000 LD_50_ BoNT/A challenge [7].

Although our previous study demonstrated the protective capacity of the Hcβtre and Hcβtre-Ad2F immunogens when delivered nasally to mice, the use of a mouse model may not be ideal when evaluating immunogens for nasal delivery to humans. For example, the mouse nasal cavity is organized to have organized nasal-associated lymphoid tissue (NALT) in the floor of the nasal cavity [8]–[12] while the NALT tissues in larger animals such as rabbits, non-human primates and humans, likely includes immune tissues distributed throughout the nasal cavity [13], [14] as well as the tonsils, adenoids and Waldeyer's ring [15], [16]. Therefore, evaluation of nasal vaccines in rabbits, animals that have a nasal cavity immune system more closely related to humans, may be an ideal animal model to evaluate the immunogenicity of vaccines proposed for nasal vaccination of humans.

Most recombinant protein vaccines lack sufficient immunogenicity and must be formulated with adjuvants to induce maximal protective immunity. We have recently reported that a novel class of vaccine adjuvants, mast cell activators, provided safe and effective vaccine adjuvant activity when delivered nasally [17] or intradermally [18] to mice. Although cholera toxin and the related labile toxin provide potent mucosal vaccine adjuvant activity, their numerous adverse effects (induction of IgE, lethal anaphylaxis, pulmonary inflammation, diarrhea, accumulation in olfactory tissues and Bell's Palsy [19]–[25]) will likely prevent their use in humans. Therefore, adjuvants that provide safe and effective adjuvant activity when delivered by a mucosal route are needed for development of mucosally-administered vaccines for human use.

In this study we evaluated our novel botulinum neurotoxin immunogens, Hcβtre and Hcβtre-Ad2F, for their ability to induce BoNT/A-neutralizing antibodies when nasally delivered to rabbits, a species whose nasal structure more closely resembles humans. Additionally, we evaluated a novel vaccine adjuvant, the mast cell activator compound 48/80 (C48/80), for its ability to enhance the immunogenicity of nasally-delivered BoNT/A subunit immunogens in rabbits. Our results demonstrate that Hcβtre-Ad2F is superior to Hcβtre for the induction of BoNT/A neutralizing antibodies after nasal delivery with adjuvant and that the mast cell activator C48/80 provides effective adjuvant activity for nasally-administered vaccines in rabbits.

## Materials and Methods

### Ethics Statement

All animal research was performed using Duke University Institutional Animal Care and Use Committee (IACUC) approved procedures under protocols A256-06-07 and A181-09-06.

### Mice and rabbits

Female BALB/c mice (Charles River/NCI) were used to perform the serum BoNT/A neutralization assay. New Zealand White (females) and Dutch Belted (females) rabbits were obtained from RSI Biotechnology (Mocksville, NC).

### Recombinant protein expression and purification

Recombinant botulinum neurotoxin type A β-trefoil (Hcβtre) and the fusion protein containing Hcβtre linked to the adenovirus type 2 fiber protein (Hcβtre-Ad2F) were produced as described previously [7]. Briefly, a synthetic gene encoding Hc BoNT/A, amino acids K1076 to L1295 (GenBank accession no. X52066), was designed for expression in the yeast, *Pichia pastoris*, and cloned into the *P. pastoris* expression vector, pPICZ B. To produce Hcβtre-Ad2F, the C-terminal region of adenovirus 2 fiber protein, spanning amino acids G378 to E582 was cloned from genomic adenovirus 2 DNA and fused to the Hcβtre plasmid. Recombinant plasmids were expressed in *P. pastoris*. Cell homogenates were cleared by centrifugation, filtered through a 1.2 µm prefilter and then through a 0.45 µm filter under vacuum. Cleared supernatants were applied to a Talon column (BD Biosciences, San Jose, CA), as per manufacturer's instruction. Purified proteins were eluted and titrated. Their quality was assessed by SDS-PAGE and Coomassie staining.

### Immunizations and sample collection

Azide-free cholera toxin (CT) was purchased from List Biological Laboratories (Campbell, CA). Compound 48/80 (C48/80) and alum were purchased from SIGMA (St. Louis, MO). Botulinum neurotoxin A (BoNT/A) toxoid and heavy chain (Hc) were purchased from Metabiologics (Madison, WI). New Zealand White female rabbits were sedated with acepromazine (1 mg/kg) and anesthetized with isoflurane (4% isoflurane at 4 liters/minute oxygen) before intranasal immunization on days 0, 14 and 28 with equimolar doses of BoNT/A Hcβtre (10 µg) or BoNT/A Hcβtre-Ad2F (20 µg) alone or combined with the adjuvants, CT (2 µg) or C48/80 (120 µg). The nasal vaccine formulation was prepared to a total volume of 250 µl with 125 µl delivered to each nostril. Rabbits were held on their backs for nasal immunization and maintained on their backs for approximately 30 seconds after nasal delivery before being returned to their cage. Rabbits were upright and conscious, although sedated, after being returned to their cage. For the alum control groups, awake rabbits were immunized intramuscularly (100 µl) with BoNT/A toxoid (10 µg) formulated with alum (350 µg) on days 0, 14 and 28. Serum was collected on days 0, 27 and 40. Dutch Belted female rabbits were sedated with acepromazine (1 mg/kg) and anesthetized with isoflurane (4% isoflurane at 4 liters/minute oxygen) before intranasal immunization on days 0, 14, 28 and 91 with equimolar doses of BoNT/A Hcβtre (10 µg) or BoNT/A Hcβtre-Ad2F (20 µg) alone or combined with the adjuvants, CT (2 µg) or C48/80 (120 µg). The nasal vaccine formulation for Dutch Belted rabbits was prepared to a total volume of 200 µl with 100 µl delivered to each nostril. Serum samples were collected days 0, 27, 41, 105 and 162. Vaginal lavage and fecal samples were collected on days 0 and 105.

### ELISA detection of antibodies specific for BoNT/A Toxoid, Hc and β-trefoil

Sera were tested for the presence of antigen-specific IgG antibodies using an ELISA protocol that utilizes the fluorescent substrate Attophos (Promega, Madison, WI) as previously reported by our group using log_2_ serum dilutions beginning at 1∶32 (1∶2^5^) [18], [26]–[28] except that the coating antigens consisted of BoNT/A toxoid, Hc or Hcβtre. Antigen-specific IgG antibodies were detected with goat anti-rabbit IgG-alkaline phosphatase (Southern Biotech, Birmingham, AL). Endpoint titers were defined as the highest reciprocal dilution of sample giving a fluorescence value 3-fold over an equally diluted naïve sample from the same animal. Log2 titers were used for statistical analysis. Samples with no detectable antibody were assigned a value 1 less than the starting log_2_ dilution for statistical analysis.

### Avidity ELISA

A modified ELISA assay was utilized to estimate the avidity of vaccine-induced anti-BoNT/A antibodies using a protocol described by others [29]–[31] with slight modifications. Day 162 serum collected from immunized Dutch Belted rabbits was diluted so that each sample produced a similar raw data anti-BoNT/A Hcβtre ELISA value and was added in duplicate to ELISA wells (as per our normal ELISA, see above) and incubated overnight at 4°C followed by washing and addition of 20 mM phosphate buffer to one well or 20 mM phosphate buffer containing 3 M ammonium thiocyanate (SIGMA, Cat. 431354) to another well followed by incubation for 15 minutes at room temperature. Following the room temperature incubation, ELISA wells were washed followed by the addition of goat anti-rabbit Ig-alkaline phosphatase (Southern Biotech, Birmingham, AL) and the assay completed as per our normal ELISA protocol. The ELISA raw data values for each sample were used to calculate the percent antibody remaining bound in the presence of 3 M ammonium thiocyanate as compared to phosphate buffer (i.e., 0 M ammonium thiocyanate).

### BoNT/A neutralization assay

A serum neutralization assay was utilized with modifications from that described by others [32] to test serum for its ability to neutralize BoNT/A. Sera were collected from Dutch Belted rabbits on days 41 and 105. Individual serum samples were diluted to the desired dilution to produce a final volume of diluted serum in 200 µl in PBS with 0.2% gelatin (SIGMA, St. Louis, MO). To the 200 µl of diluted serum was added 200 µl PBS/gelatin containing 20 LD_50_ Botulinum Neurotoxin A (Metabiologics, Madison, Wisconsin). The serum and toxin mixture were incubated at room temperature for 1 hour before 200 µl of the mixture (containing 10 LD_50_ BoNT/A) was injected intraperitoneally into naïve, female BALB/c mice. Mice were monitored after 2 and 6 hours and then daily for signs of morbidity, including difficulty breathing and lack of mobility. Mice exhibiting morbidity were euthanized with Duke IACUC approved methods.

### Statistical Analysis

Log_2_ ELISA antibody titers and BoNT/A neutralization titers were compared by ANOVA, followed by Tukey's multiple comparison method. The Mann-Whitney test was used to compare neutralizing antibody titers grouped by antigen (Hcβtre + adjuvant vs Hcβre-Ad2F + adjuvant) to determine if their were significant differences between adjuvanted Hcβtre or Hcβtre-Ad2F in their ability to induce BoNT/A neutralizing antibody. The Mann-Whitney test was also used to compare antibody avidity (% antibody bound in the presence of 3 M ammonium thiocyanate) between serum with no BoNT/A neutralization activity and serum with detectable BoNT/A neutralization activity. Significant differences were defined as p<0.05.

## Results

### Ad2F enhances the immunogenicity of BoNT/A Hcβtre in New Zealand White rabbits after intranasal immunization with cholera toxin or the mast cell activator adjuvant compound 48/80

Our previous study [7] demonstrated that a fusion protein consisting of the botulinum neurotoxin A Hcβtre domain and the adenovirus type 2 fiber protein (Hcβtre-Ad2F; **Figure 1**) exhibited immunogenicity that was superior to that observed for Hcβtre when both were used as nasal vaccine immunogens. To determine if Hcβtre-Ad2F exhibited immunogenicity superior to Hcβtre after nasal delivery to a host with a nasal cavity similar to humans [13], [14], immunogenicity studies were performed in rabbits. This study was also performed to evaluate the ability of a novel class of vaccine adjuvants, mast cell activators [17], [18] to provide adjuvant activity in rabbits. Female New Zealand White rabbits (3–6 rabbits per group) were nasally immunized on days 0, 14 and 28 with equimolar doses of Hcβtre (10 µg) or Hcβtre-Ad2F (20 µg) alone or combined with the adjuvants, CT (2 µg) or C48/80 (120 µg). To compare the immunogenicity and antigenicity of the Hcβtre immunogens to other forms of BoNT/A immunogens, BoNT/A toxoid and BoNT/A Hc were used as control immunogens. Rabbits were immunized with BoNT/A toxoid (10 µg) + alum intramuscularly on days 0, 14 and 28 while BoNT/A Hc (20 µg) combined with CT (2 µg) or C48/80 (120 µg) was delivered nasally on days 0, 14 and 28. Serum was collected on days 0, 28 and 40 and tested for IgG specific for BoNT/A toxoid, recombinant BoNT/A Hc or the β-trefoil domain of BoNT/A Hc (Hcβtre) (**Figure 1**). Serum titers were calculated and reported as endpoint geometric means for each group.

**Figure 2:**
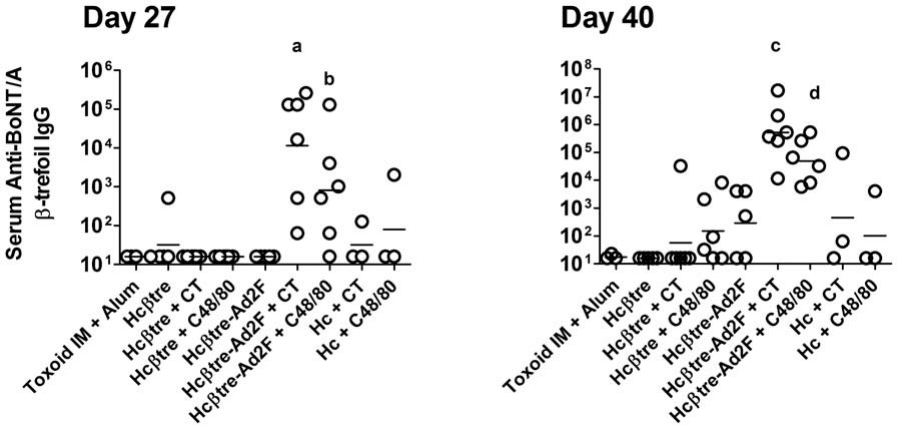
Ad2 fiber protein enhances the nasal immunogenicity of BoNT/A β-trefoil in NZW rabbits. Female NZW rabbits were immunized on days 0, 14 and 28 with the indicated vaccine formulation. Intramuscular immunization with 10 µg of BoNT/A toxoid combined with alum (n = 4) served as a control. BoNT/A Hcβtre (10 µg) was nasally delivered in the absence of adjuvant (n = 5) or combined with CT (2 µg; n = 6) or C48/80 (120 µg; n = 6). BoNT/A Hcβtre-Ad2F (20 µg) was delivered nasally in the absence of adjuvant (n = 5) or combined with CT (2 µg; n = 6) or C48/80 (120 µg; n = 6). BoNT/A Hc (20 µg) was delivered nasally combined with CT (2 µg; n = 3) or C48/80 (120 µg; n = 3). Serum samples collected on day 27 and day 40 were tested for the presence of anti-BoNT/A β-trefoil IgG by ELISA. Serum antibody titers were compared between groups by ANOVA followed by Tukey's multiple comparison test (GraphPad, Prism). **a**: serum anti-BoNT/A β-trefoil IgG titers significantly greater than those induced by intramuscular immunization with toxoid, nasal immunization with Hcβtre, nasal immunization with Hcβtre + CT, nasal immunization with Hcβtre + C48/80, nasal immunization with Hcβtre-Ad2F, nasal immunization with Hc + CT and nasal immunization with Hc + C48/80. **b**: serum anti-BoNT/A β-trefoil IgG titers significantly greater than those induced by nasal immunization with Hcβtre + CT and nasal immunization with Hcβtre + C48/80. **c**: serum anti-BoNT/A β-trefoil IgG titers significantly greater than those induced by intramuscular immunization with toxoid, nasal immunization with Hcβtre, nasal immunization with Hcβtre-Ad2F, nasal immunization with Hcβtre + CT, nasal immunization with Hcβtre + C48/80, nasal immunization with Hc + CT and nasal immunization with Hc + C48/80. **d**: serum anti-BoNT/A β-trefoil IgG titers significantly greater than those induced by intramuscular immunization with toxoid, nasal immunization with Hcβtre, nasal immunization with Hcβtre + CT, immunization with Hcβtre + C48/80, nasal immunization with Hcβtre-Ad2F and nasal immunization with Hc + C48/80.

Nasal immunization with Hcβtre-Ad2F immunogens formulated with CT or C48/80 as adjuvants induced the highest serum anti-Hcβtre IgG titers at Day 27 and Day 40 (**Figure 2**). At Day 27, nasal immunization with Hcβtre-Ad2F + CT induced a serum anti-BoNT/A βtre IgG titer of 1∶11,585 while nasal immunization with Hcβtre-Ad2F + C48/80 induced a serum anti-BoNT/A Hcβtre IgG titer of 1∶813; the only groups with serum anti-Hcβtre significantly greater than those induced by other vaccines (**Figure 2**). Despite nasal immunization with an equimolar dose of Hcβtre adjuvanted with CT or C48/80, Hcβtre immunogens failed to induce significantly elevated serum anti-BoNT/A IgG titers (**Figure 2**). Nasal immunization with recombinant BoNT/A Hc adjuvanted with CT or C48/80 also failed to induce significantly elevated serum anti-BoNT/A Hcβtre IgG titers. Of particular interest was the observation that intramuscular immunization with BoNT/A toxoid adjuvanted with alum failed to induce serum IgG antibodies that recognized the BoNT/A Hcβtre domain (**Figure 2**). Similar results were observed at day 40 with serum anti-BoNT/A Hcβtre IgG titers of 1∶524,288 and 1∶49,097 for rabbits nasally immunized with Hcβtre-Ad2F + CT or C48/80, respectively. Serum titers induced by C48/80 were not significantly different than those induced by CT. Our results demonstrate that Hcβtre-Ad2F, an immunogen designed to contain a mucosal targeting component, provided nasal immunogenicity that was superior to immunogens lacking the mucosal targeting domain. Additionally, the mast cell activator C48/80 provided significant adjuvant activity after nasal delivery to rabbits.

**Figure 3:**
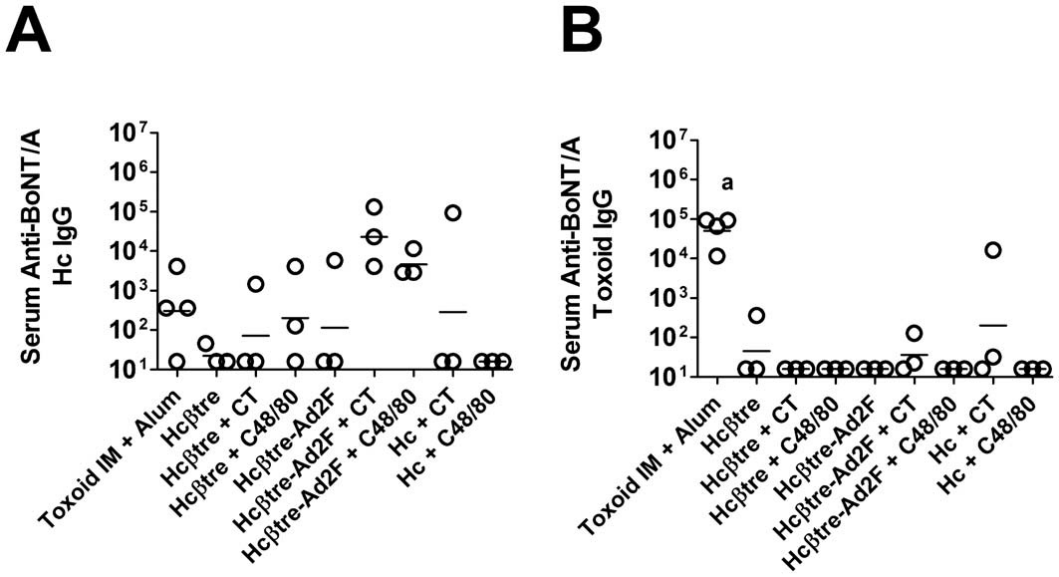
BoNT/A Hcβtre immunogens induce antibodies that recognize epitopes distinct from those induced by BoNT/A toxoid. Day 40 sera from a subset of rabbits included in Figure 1 were tested for the presence of antibodies specific for BoNT/A Hc or BoNT/A toxoid by ELISA. **a**: serum anti-BoNT/A toxoid IgG titers significantly greater than those induced by all other groups. There were no other significant differences between groups.

Recombinant BoNT/A Hc immunogens are currently in development as next generation BoNT vaccines [5], [33]. Despite the lack of immunogenicity of Hc when used as a nasal vaccine, as measured by the induction of anti-Hcβtre IgG titers, it is possible that Hc immunogens induce antibodies that recognize epitopes outside of the Hcβtre domain. We therefore tested day 40 serum collected from the rabbit groups described in Figure 2 for the presence of anti-BoNT/A Hc antibodies by ELISA (**Figure 3A**). The anti-BoNT/A Hc serum IgG titers at day 40 were similar to the anti-BoNT/A Hcβtre IgG responses with the highest anti-Hcβtre IgG titers in rabbits immunized intranasally with Hcβtre-Ad2F + CT (1∶23,170) or Hcβtre-Ad2F + C48/80 (1∶4,598). Due to the variability of the anti-BoNT/A Hc antibody responses, there were no significant differences between any of the groups. These results support the findings discussed in Figure 2 and demonstrate that nasal immunization with Hcβtre-Ad2F immunogens and adjuvant (CT or C48/80) induced maximal anti-BoNT/A Hc antibody responses that were at least 10-fold greater than antibody responses induced by any other vaccine group tested.

Since the current investigational vaccine for botulinum neurotoxin is a toxoid [5], [33] and the toxoid may be antigenically distinct from the recombinant immunogens [34], [35], day 40 serum samples were also tested for the presence of antibodies that recognize BoNT/A toxoid (**Figure 3B**). As expected, intramuscular immunization with BoNT/A toxoid + alum induced significantly increased serum anti-BoNT/A toxoid IgG titers (1∶50,535) that were significantly greater than all other vaccine groups tested. In agreement with published literature [34], [35], our results demonstrate that the antigenicity of BoNT/A toxoid is significantly different than recombinant forms of immunogens since immunization with BoNT/A toxoid induced antibodies able to recognize toxoid but not BoNT/A Hcβtre or Hc.

### Ad2F enhances β-trefoil immunogenicity in Dutch Belted rabbits after intranasal immunization with cholera toxin or the mast cell activator adjuvant C48/80

**Figure 4:**
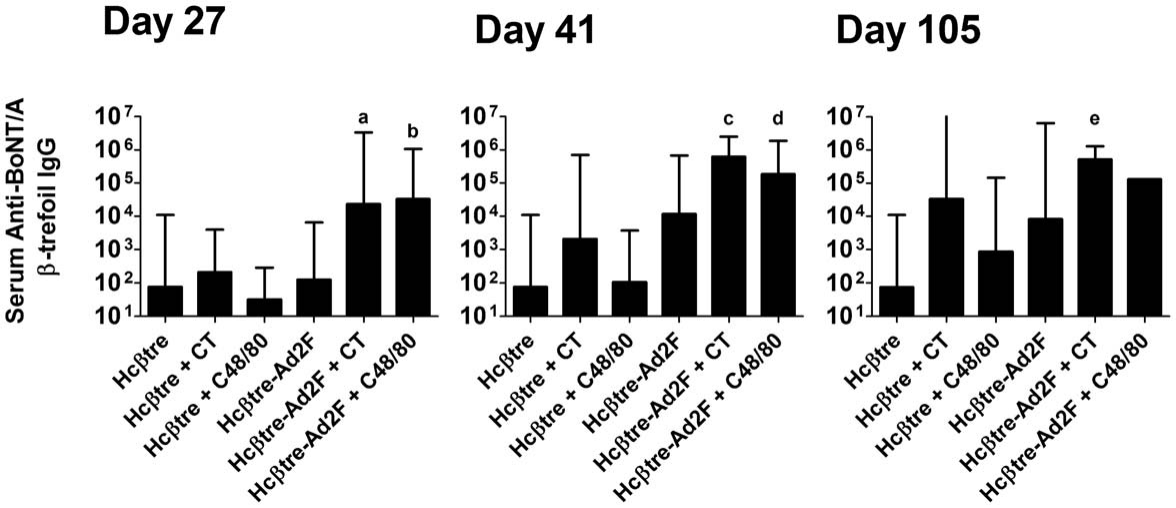
Ad2 fiber protein enhances the nasal immunogenicity of BoNT/A β-trefoil in Dutch Belted rabbits. Female Dutch Belted rabbits (4 per group) were nasally immunized on days 0, 14, 28 and 91 with the indicated vaccine formulation. Serum collected on days 27, 41 and 105 was tested for the presence of anti-BoNT/A β-trefoil IgG by ELISA. **a**: serum anti-BoNT/A β-trefoil IgG titers significantly greater than those induced by nasal immunization with Hcβtre or nasal immunization with Hcβtre + C48/80. **b**: serum anti-BoNT/A β-trefoil IgG titers significantly greater than those induced by nasal immunization with Hcβtre, nasal immunization with Hcβtre + C48/80 or nasal immunization with Hcβtre-Ad2F. **c**: serum anti-BoNT/A β-trefoil IgG titers significantly greater than those induced by nasal immunization with Hcβtre, nasal immunization with Hcβtre + CT, nasal immunization with Hcβtre + C48/80. **d**: serum anti-BoNT/A β-trefoil IgG titers significantly greater than those induced by nasal immunization with Hcβtre or nasal immunization with Hcβtre + C48/80. **e**: serum anti-BoNT/A β-trefoil IgG titers significantly greater than those induced by nasal immunization with Hcβtre.

To determine if the superior immunogenicity of Hcβtre-Ad2F and the adjuvant activity of C48/80 could be confirmed in a second rabbit strain, we repeated the intranasal immunization protocol using BoNT/A Hcβtre or Hcβtre-Ad2F ± CT or C48/80 in Dutch Belted rabbits. Four rabbits per group were immunized on days 0, 14, 28 and 91 with the same molar doses of Hcβtre (10 µg) or Hcβtre-Ad2F (20 µg) alone or combined with CT (2 µg) or C48/80 (120 µg). Sera collected on days 27, 41 and 105 were evaluated for the presence of β-trefoil specific IgG antibodies by ELISA (**Figure 4**). On day 27, the only groups with significantly increased serum anti-BoNT/A Hcβtre IgG titers were rabbits nasally immunized with Hcβtre-Ad2F + CT (1∶23,170) or Hcβtre-Ad2F + C48/80 (1∶32,768). These results demonstrate that the addition of Ad2F as a mucosal targeting ligand when combined with adjuvant (CT or C48/80) enhanced the immunogenicity of the Hcβtre immunogen and improved the induction of serum anti-Hcβtre IgG responses after nasal vaccination. The serum anti-Hcβtre IgG titers induced by nasal immunization with Hcβtre-Ad2F + C48/80 were significantly greater than titers induced by nasal immunization with Hcβtre-Ad2F alone (p<0.05) demonstrating the mucosal adjuvant activity of C48/80 (**Figure 4**).

The day 41 serum anti-Hcβtre IgG profile (2 weeks after the day 28 booster dose) was similar to the day 27 responses and the only groups that generated significantly increased serum anti-Hcβtre IgG titers were Hcβtre-Ad2F + CT (1∶623,487) or Hcβtre-Ad2F + C48/80 (1∶185,364). The serum anti-Hcβtre IgG titers induced by nasal immunization with Hcβtre-Ad2F + CT were significantly greater than anti-Hcβtre IgG titers induced by nasal immunization with Hcβtre, Hcβtre + CT, Hcβtre + C48/80 (**Figure 4**, p<0.05). Serum anti-Hcβtre IgG titers induced by nasal immunization with Hcβtre-Ad2F + C48/80 were significantly greater than anti-Hcβtre IgG titers induced by nasal immunization with Hcβtre or Hcβtre + C48/80 (**Figure 4**, p<0.05). For each vaccine formulation tested (i.e., antigen alone, antigen + CT or antigen + C48/80), the presence of the Ad2F domain increased the induction of serum anti-Hcβtre IgG antibody titers. For example, nasal immunization with Hcβtre alone induced a serum anti-Hcβtre IgG titer of 1∶76 while nasal immunization with Hcβtre-Ad2F induced a serum titer of 1∶11,585. When administered with CT, nasal Hcβtre induced a serum titer of 1∶2,048 while nasal immunization with CT and Hcβtre-Ad2F induced a serum titer of 1∶623,487. Finally, when administered with C48/80, nasal immunization with Hcβtre induced a serum anti-Hcβtre IgG titer of 1∶108 while nasal immunization with Hcβtre-Ad2F induced a serum titer of 1∶185,364. Our results demonstrate that the presence of Ad2F greatly enhanced the immunogenicity of Hcβtre when used as a nasal vaccine immunogen.

Serum collected on day 105 and tested for the presence of anti-Hcβtre IgG titers demonstrated that the anti-Hcβtre IgG titers had increased in all groups although the serum titers induced by nasal immunization with Hcβtre-Ad2F adjuvanted with CT or C48/80 remained the highest (1∶524,288 and 1∶131,072, respectively).

### Ad2F as a mucosal targeting ligand enhances the induction of BoNT/A neutralizing antibodies after nasal immunization with Hcβtre immunogens

We sought to determine whether the antibodies generated by our Hcβtre vaccines could protect against BoNT/A toxin challenge and whether the addition of Ad2F enhanced the induction of BoNT/A neutralizing antibodies. A mouse neutralization assay [32] was used to measure BoNT/A neutralizing antibodies in day 41 serum (after three doses of vaccine). None of the vaccines tested induced toxin-neutralizing serum antibodies at day 41(data not shown).

**Figure 5:**
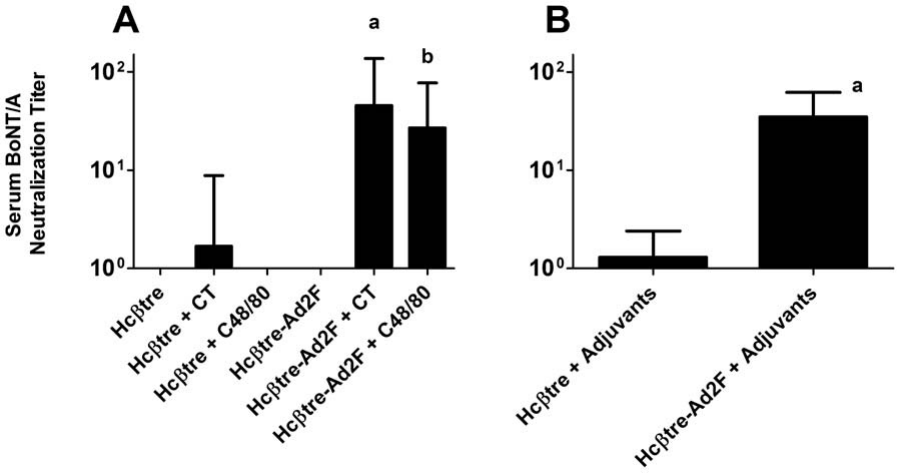
Ad2 fiber protein enhances the induction of BoNT/A-neutralizing antibodies in nasally immunized rabbits. Female Dutch Belted rabbits (4 per group) were nasally immunized on days 0, 14, 28 and 91 with the indicated vaccine formulation. **A**. Serum collected on day 105 was tested for the presence of BoNT/A-neutralizing antibodies using a mouse neutralization assay. **a**: serum BoNT/A-neutralizing activity significantly greater than the neutralizing activity measured in rabbits nasally immunized with Hcβtre, Hcβtre + CT, Hcβtre + C48/80 or Hcβtre-Ad2F; ANOVA and Tukey's multiple comparison, p<0.05. **b**: serum BoNT/A-neutralizing activity significantly greater than the neutralizing activity measured in rabbits nasally immunized with Hcβtre, Hcβtre + CT, Hcβtre + C48/80 or Hcβtre-Ad2F; ANOVA and Tukey's multiple comparison, p<0.05. **B**. Hcβtre-Ad2F is superior to Hcβtre for the induction of BoNT/A neutralizing antibodies when delivered with CT or C48/80 as adjuvants. **a**: p = 0.0006, Mann-Whitney test.

Serum collected on day 105 (after a total of 4 vaccinations) was also tested for the presence of BoNT/A neutralizing antibodies. One rabbit nasally immunized with Hcβtre + CT had measurable BoNT/A neutralizing antibodies (titer of 1∶8) while all other rabbits in that group and none of the rabbits nasally immunized with Hcβtre, Hcβtre + C48/80 or Hcβtre-Ad2F had measurable serum BoNT/A neutralizing antibodies. Rabbits nasally immunized with Hcβtre-Ad2F + CT had a geometric mean serum BoNT/A neutralizing titer of 1∶45.25 while rabbits nasally immunized with Hcβtre-Ad2F + C48/80 had a serum BoNT/A neutralizing titer of 1∶26.91 (**Figure 5A**). Serum BoNT/A neutralizing antibody titers induced by nasal immunization with Hcβtre-Ad2F + CT or Hcβtre-Ad2F + C48/80 were significantly greater than the neutralizing antibody titers measured in rabbits nasally immunized with Hcβtre, Hcβtre + CT, Hcβtre + C48/80 or Hcβtre-Ad2F; ANOVA and Tukey's multiple comparison (**Figure 5A** **, p<0.05**). By combining serum BoNT/A neutralizing antibody titers induced by nasal immunization with Hcβtre + CT or C48/80 (**Figure 5B**, Hcβtre + adjuvants) and comparing their serum neutralization titer to those induced by nasal immunization with Hcβtre-Ad2F + CT or C48/80 (**Figure 5B**, Hcβtre-Ad2F + adjuvants), we determined that the presence of Ad2F significantly improved the induction of BoNT/A neutralizing antibodies (p = 0.0006), regardless of the adjuvant used. These results demonstrate that the use of Ad2F as a mucosal targeting ligand as well as the use of adjuvant (CT or C48/80) was required for effective induction of serum BoNT/A neutralizing antibody responses. Additionally, C48/80 provided effective nasal adjuvant activity in rabbits that was comparable to that provided by CT.

### Antibody affinity correlates with protective immunity

**Figure 6:**
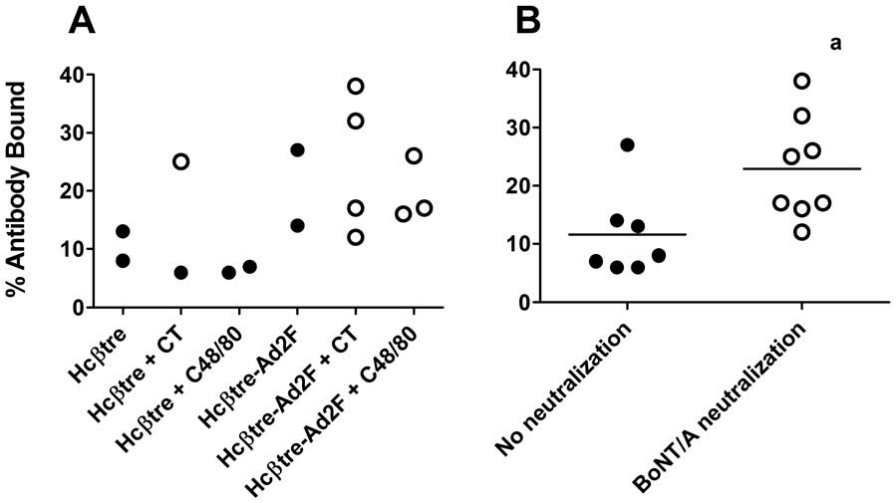
Adjuvants enhance the avidity of anti-BoNT/A β-trefoil IgG antibodies. The relative avidity of anti-BoNT/A IgG serum antibodies was measured using day 162 serum using a modified ELISA as described in Materials and Methods. **A**. The percent antibody bound in the ELISA in the presence of 3 M ammonium thiocyanate is indicated for individual serum samples that had measurable anti-β-trefoil IgG. A higher percent antibody bound represents a greater relative avidity for vaccine-induced, antigen-specific antibody. •: represents serum anti-β-trefoil IgG with no detectable BoNT/A neutralizing activity. ○: represents serum anti-β-trefoil IgG with detectable BoNT/A neutralizing activity. **B**. Average percent antibody bound when samples were organized by the neutralization capacity of the serum. Vaccine-induced BoNT/A neutralizing antibody has significantly greater avidity than non-neutralizing antibody. **a**: % antibody bound significantly greater than in the no neutralization group. Two-tailed Mann Whitney, p = 0.0239.

The binding avidity of antibodies for their antigen may influence their neutralization capacity [36]-[38]. To determine if antibody affinity for Hcβtre correlated with its neutralization activity, we measured the relative avidity of serum anti-Hcβtre Ig using a thiocyanate elution ELISA similar to that described by others [29]–[31]. In this assay, the amount of antigen-specific antibody remaining bound in the presence of 3 M ammonium thiocyanate, calculated as a percentage of antibody bound in the absence of ammonium thiocyante, is used to estimate the relative avidity of the antibodies. The percentage of antibody remaining bound in the presence of 3 M ammonium thiocyanate increases as the avidity of the antibody increases. The relative avidity of anti-Hcβtre Ig in Day 162 serum was tested for all samples that had measurable anti-Hcβtre serum antibodies (**Figure 6**). Due to the variation in the relative avidities measured in the various groups, there was no significant difference in antibody avidity between the different groups (**Figure 6A**). However, grouping of the serum samples based on their neutralization capacity demonstrated that serum samples with BoNT/A neutralization activity had an anti-Hcβtre Ig relative avidity that was greater than that in samples without BoNT/A neutralization activity (p = 0.0239; **Figure 6B**).

## Discussion

In this study we have demonstrated that the β-trefoil domain of BoNT/A (Hcβtre) can be used as an immunogen for nasal vaccination in rabbits to induce serum antibodies that protect against BoNT/A challenge. Our results show that production of the Hcβtre immunogen as a fusion protein with the mucosal targeting ligand, Ad2F, enhanced its immunogenicity and the induction of Hcβtre-specific, BoNT/A-neutralizing antibodies. Additionally, the mast cell activator adjuvant C48/80 was as effective as CT for the induction of BoNT/A-neutralizing antibodies when used as a nasal vaccine adjuvant in rabbits. These results confirm similar observations in mice [7], [17].

Adjuvants and vaccination regimens suitable for nasal immunization are desirable for a number of reasons. First, one goal of the World Health Organization is to have vaccines administered needle-free [39]–[43], such as nasal immunization, since the re-use of needles in developing countries contributes to the transmission of infectious diseases. Second, nasal immunization offers a beneficial alternative for patients with needle-phobia and may prevent their avoidance to vaccination [44]–[46]. Third, nasal immunization has the ability to induce antigen-specific secretory IgA (S-IgA) responses in mucosal secretions, while parenterally delivered vaccines rarely induce S-IgA [47]–[49]. A major benefit of mucosal immunization is the presence of pathogen-specific S-IgA in mucosal secretions offering an added layer of protection against pathogens that primarily infect via a mucosal surface. Such capacity to induce vaccine-specific S-IgA as well as the ease of needle-free immunization warrants continued research to optimize adjuvants and antigen formulations for nasal subunit vaccines.

The form of antigen used as a vaccine immunogen significantly influences the immune responses induced. To evaluate the performance of Hcβtre immunogens as compared to other BoNT/A immunogens, we utilized BoNT/A toxoid and BoNT/A Hc immunogens as controls for subcutaneous or intranasal immunization, respectively. Of all immunogens tested, only Hcβtre-Ad2F combined with adjuvant (CT or C48/80) induced significantly elevated serum anti-Hcβtre IgG titers. Despite using BoNT/A Hc as an immunogen at the same dose as Hcβtre-Ad2F and formulated with CT or C48/80 as adjuvants, Hc did not induce significantly elevated serum anti-Hcβtre or anti-Hc IgG titers, even though the Hcβtre domain represents a significant portion of the Hc immunogen. BoNT/A toxoid delivered intramuscularly with alum induced significantly elevated serum anti-BoNT/A toxoid IgG antibodies while inducing minimal serum IgG antibodies specific for Hcβtre or Hc. This result confirms published reports describing the altered antigenicity of BoNT/A toxoid as compared to native immunogens [34], [35] and supports the use of recombinant subunit immunogens as next generation BoNT vaccines.

The immunization regimens utilized in this study used relatively low antigen doses. We selected a dose of 10 µg of Hcβtre per vaccination and an equimolar dose of 20 µg Hcβtre-Ad2F to increase the sensitivity of the experiment to determine if the use of the mucosal targeting ligand Ad2F was able to significantly enhance the immunogenicity of Hcβtre. In our previous mouse study, Hcβtre-Ad2F exhibited superior nasal immunogenicity when compared to Hcβtre based on the induction of ELISA binding antibodies but both immunogens induced BoNT/A neutralizing antibody responses when used at 25 µg of Hcβtre per dose and 50 µg of Hcβtre-Ad2F per dose [7]. In the present study, nasal immunization with Hcβtre-Ad2F adjuvanted with CT or C48/80 induced serum neutralization titers in Dutch belted rabbits that ranged from 1∶16 to 1∶128 with each rabbit being immunized with a total of 80 µg of Hcβtre-Ad2F combined with adjuvant. The neutralization assay tested diluted serum for its ability to neutralize 10 LD_50_ of BoNT/A using a mouse neutralization assay. Others have reported that immunization of rabbits intradermally with a total dose 750 µg of BoNT/A Hc (residues 871-1295) adjuvanted with complete (priming) and incomplete (booster doses) Freund's adjuvant in five doses induced serum BoNT/A neutralizing antibodies with a neutralization capacity that ranged from 5×10^5^ to 7×10^5^ mouse LD_50_ per mL serum [32]. Given in these terms, our adjuvanted Hcβtre-Ad2F vaccine exhibited a BoNT/A neutralization capacity ranging from 1.6×10^3^ to 1.28×10^4^ mouse LD_50_ per ml of serum while using only 80 µg of total antigen and nasal delivery. One rabbit immunized with a Hcβtre and CT had a serum BoNT/A neutralization capacity of 800 mouse LD_50_ per ml of serum demonstrating the ability of Hcβtre to induce BoNT/A neutralizing antibodies. Our use of higher doses of Hcβtre or Hcβtre-Ad2F would likely have induced more potent BoNT/A neutralizing antibody responses.

The exact mechanism utilized by Ad2F to augment the immunogenicity of nasally-delivered Hcβtre is not clear. We originally selected Ad2F as a mucosal targeting ligand and demonstrated its ability to mediate antigen binding at the epithelial surface in the mouse nasal cavity [7]. Therefore, it is possible that Hcβtre-Ad2F immunogens bind to the epithelial surface after nasal delivery to rabbits resulting in enhanced antigen retention within the nasal cavity allowing more antigen to be available for induction of Hcβtre-specific immune responses. This conclusion is supported by recent studies in our group demonstrating that nasal vaccination regimens in rabbits that enhance retention of the vaccine within the nasal cavity enhance the induction of antigen-specific serum antibodies [50]. Studies in the mouse that demonstrate superior induction of BoNT/A neutralizing antibodies when using vaccine formulations that result in continued adherence of the vaccine to the nasal epithelium also support this conclusion [51]. Although antigen retention within the nasal cavity may contribute to the enhanced immunogenicity of Hcβtre-Ad2F immunogens, the ability of Ad2F to induce cytokine and chemokine expression after binding to its receptor, the coxsackie-adenovirus receptor (CAR), may also contribute to the superior immunogenicity of Hcβtre-Ad2F [52].

Induction of BoNT/A-neutralizing antibodies required the use of an adjuvant. We utilized CT as a classic mucosal vaccine adjuvant to determine if the mast cell activator C48/80 [17] provided effective adjuvant activity in the rabbit comparable to that provided by CT. C48/80 provided effective adjuvant activity that was comparable to that provided by CT after nasal immunization with Hcβtre-Ad2F. We have previously reported the C48/80 provided effective adjuvant activity in mice while not inducing antigen-specific IgE responses or other adverse effects such as induction of anaphylactic reactions [17], [18]. Although antigen-specific IgE responses were not monitored in this rabbit study, the use of C48/80 was not associated with the induction of adverse effects such as immediate immunization site reactions or hypersensitivity reactions. While additional studies are needed to thoroughly evaluate the preclinical toxicity of C48/80, no adverse effects have been observed to date. Our results are the first to demonstrate that mast cell activators provide safe and effective mucosal vaccine adjuvant activity in rabbits.

Many factors such as antibody concentration, specificity and avidity may contribute to the biological activity of vaccine-induced antibodies. Attempts at mapping linear epitopes recognized by Hcβtre and Hcβtre-Ad2F vaccine-induced antibodies were unsuccessful (data not shown) suggesting that the antibodies induced by the Hcβtre immunogens recognize conformational epitopes. By measuring the relative avidity of anti-Hcβtre serum Ig with the use of a modified ELISA [29]–[31] that tests serum at similar antibody concentrations, we determined that serum samples with BoNT/A neutralization activity exhibited a relative binding avidity that was significantly higher than the relative avidity of non-neutralizing antibodies. Our results are in agreement with observations that the biological activity of antibodies is positively correlated with their binding avidity for their specific antigen [37], [38], [53]–[55]. Induction of high avidity, BoNT/A-neutralizing antibodies required the use of adjuvants (CT or C48/80) and future studies will work to optimize vaccine formulations and adjuvants for their ability to induce high-avidity antibodies.

Nasal immunization with Hcβtre or Hcβtre-Ad2F in Dutch belted rabbits induced minimal vaginal or fecal anti-Hcβtre IgA responses (data not shown). Although we have demonstrated in mouse models that nasal vaccination is an effective method of immunization for the induction of antigen-specific mucosal IgA [7], [50], [56]–[59], our nasal immunization studies in non-human primates [59], [60] and rabbits [50] have failed to consistently induce mucosal IgA responses in fecal samples or vaginal secretions. Our results in rabbits are supported by published reports from others that also failed to induce significant mucosal IgA responses after nasal immunization [61], [62]. Our use of low antigen doses may have contributed to the poor induction of mucosal IgA in rabbits after nasal immunization with Hcβtre immunogens since antigen dose has been demonstrated to influence the induction of mucosal IgA responses after nasal immunization [63]. Collectively, these results suggest that the conditions required for induction of mucosal IgA in non-rodents requires further studies.

While the live-attenuated influenza vaccine (LAIV) provides a convincing example that nasally-administered vaccines may be approved by the U.S. Food and Drug Administration (FDA) and are able to safely induce the desired immune responses in humans [64]–[66], additional pre-clinical studies are needed to develop nasal subunit vaccines for use in humans. For example, the potential for vaccine-induced inflammation in the lungs is a significant concern when considering the development of nasally-administered vaccines for use in humans since murine nasal immunization studies have demonstrated the induction of antigen-specific IgE and airway inflammation when potent adjuvants such as cholera toxin are used [22], [67]. Clear advantages of using our vaccine formulation are the lack of vaccine-induced IgE or anaphylactic reactions by C48/80 when used as a nasal vaccine adjuvant in mice [17] and the lack of sensitivity reactions in rabbits. To avoid potential lung inflammation, nasal immunization methods must include in its design a means to retain the vaccine in the nares, while simultaneously preventing subsequent deposition into the lungs. This can be accomplished by limiting the vaccine to the upper respiratory tract by nasal sprays that deliver large droplets [67] or the use of dry powder vaccine formulations with large particles (i.e., approximately 10 µm) that are directed only to the upper respiratory tract and unable to reach the lung [68]. Since humans are repeatedly exposed to natural upper respiratory tract infections that induce inflammation and antigen-specific immune responses with no obvious deleterious effects to the host, it seems unlikely that adjuvant-dependent or antigen-specific immune responses induced by nasal immunization would initiate adverse effects in the host. Additional preclinical studies are needed to evaluate the safety and toxicity of nasally-delivered subunit vaccines.

To summarize, recombinant BoNT/A immunogens utilizing the Hcβtre domain are effective immunogens that contain BoNT/A neutralizing epitopes. When expressed as a fusion protein with Hcβtre, the mucosal targeting ligand Ad2F significantly improved the nasal immunogenicity of Hcβtre and enhanced the induction of BoNT/A neutralizing antibodies with increased avidity when delivered with adjuvants. The chemical mast cell activator C48/80 provided adjuvant activity for nasally administered Hcβtre-Ad2F that was comparable to the adjuvant activity of cholera toxin. Collectively, our results suggest that the use of the mucosal targeting ligand Ad2F and the novel adjuvant C48/80 are effective methods to augment the immunogenicity of nasally-delivered subunit immunogens for the induction of antibodies with increased avidity and biological activity.
